# Relationship between clinician documented blast exposure and pulmonary function: a retrospective chart review from a national specialty clinic

**DOI:** 10.1186/s12931-022-02071-0

**Published:** 2022-06-10

**Authors:** Jennifer H. Therkorn, Sean Hu, Anays M. Sotolongo, Israel C. Christie, Tianshi David Wu, William W. Van Doren, Venkata Siva Sai Sujith Sajja, Nisha Jani, Jacquelyn C. Klein-Adams, Drew A. Helmer, Michael J. Falvo

**Affiliations:** 1 Airborne Hazards and Burn Pits Center of Excellence, War Related Illness and Injury Study Center, VA New Jersey Health Care System, 385 Tremont Ave, East Orange, NJ 07018 USA; 2 New Jersey Medical School, Rutgers, The State University, Newark, NJ USA; 3 Section of Health Services Research, Baylor College of Medicine, Houston, TX USA; 4 Center for Innovations in Quality, Effectiveness, and Safety, Michael E. DeBakey VA Medical Center, Houston, TX USA; 5 Section of Pulmonary, Critical Care, and Sleep Medicine, Baylor College of Medicine, Houston, TX USA; 6 Blast Induced Neurotrauma Branch, Walter Reed Army Institute of Research, Silver Spring, MD USA

## Abstract

**Background:**

Service member exposure to explosive blast overpressure waves is common with considerable attention to traumatic brain injury (TBI) and neuropsychological sequalae. Less is known about the impacts on the respiratory system, particularly long-term effects, despite vulnerability to overpressure. Using a national registry, we previously observed an independent relationship between self-reported blast exposure and respiratory symptoms; however, the impact on objective measures of pulmonary function is poorly understood.

**Methods:**

307 Veterans referred to our national specialty center for post-deployment health concerns underwent a comprehensive multi-day evaluation that included complete pulmonary function testing (PFT), occupational and environmental medicine history, neuropsychological or psychological evaluation. We developed an a priori chart abstraction process and template to classify Veterans into blast exposure groups: (1) none, (2) single-mild, or (3) multiple-mild. This template focused primarily on clinician documented notes of blast related TBI that were used as proxy for blast overpressure injury to thorax. PFT variables characterizing flow (FEV_1_%; %∆FEV_1_), volume (TLC%), diffusion (DL_CO_%) and respiratory mechanics (forced oscillometry) were selected for analysis.

**Results:**

Veterans (40.5 ± 9.7 years; 16.3% female) were referred 8.6 ± 3.6 years after their last deployment and presented with considerable comorbid conditions and health problems (e.g., 62% post-traumatic stress, 55% dyspnea). After chart abstraction, Veterans were assigned to none (n = 208), single mild (n = 52) and multiple mild (n = 47) blast exposure groups. Among the blast exposed, clinicians documented 73.7% were < 50 m from the blast and 40.4% were physically moved by blast. PFT outcome measures were similar across all groups (*p* value range: 0.10–0.99).

**Conclusions:**

In this referred sample of deployed Veterans, PFT measures of flow, volume, diffusion, and respiratory mechanics were not associated with clinician documented blast exposure per the retrospective chart abstraction methodology applied. Yet, these clinical findings suggest future research should determine and assess distinction between Veteran recollections of perceived blast experiences versus overpressure wave exposure to the respiratory system.

**Supplementary Information:**

The online version contains supplementary material available at 10.1186/s12931-022-02071-0.

## Introduction

Approximately 8 in 10 combat injuries in recent conflicts have an explosive blast etiology [1] which includes air blast wave propagation from improvised explosive devices (IED). IEDs are a distinguishing feature of the conflicts in Iraq and Afghanistan [2] and well-recognized to induce traumatic brain injury (TBI) [3]. However, organ systems other than the brain, such as the lungs, ears and the gastrointestinal tract, are uniquely vulnerable to blast overpressure (i.e., rapidly changing pressure gradient) yet long term outcomes and effects of exposures on these systems have been understudied relative to TBI [4]. Recent studies have begun to investigate the association between blast overpressure during deployment and pulmonary outcomes. Pugh and colleagues conducted a retrospective review of Veterans Affairs (VA) medical encounters between 2003 and 2011 and observed an increasing prevalence of chronic obstructive pulmonary disease and asthma among those deployed in support of combat operations in Iraq and Afghanistan [5]. In their analyses, an association between TBI and chronic lung disease was observed; the authors cautiously interpreted this association as potential evidence of a role for blast exposure with TBI diagnosis serving as a proxy for IED blast exposure.

Using data from the national VA Airborne Hazards and Open Burn Pit Registry, we previously observed an independent association (adjusted odds ratio 1.66, 95% CI 1.5–1.7) between IED blast exposure and cardiopulmonary symptoms, even after adjustment for potential confounding factors such as burn pit smoke exposure and smoking [6]. This study was limited by reliance on a dichotomous, self-reported representation of blast exposure, and did not assess physiological outcomes. An alternative methodology to classify blast exposure was recently described by Zell-Baran et al. [7] who developed a ‘blast exposure intensity score’ that was the sum of the product of deployment length (months) and frequency (days·month^−1^) of IED blasts and controlled detonations. Investigators observed an unadjusted association between their blast exposure severity score and lung clearance index (marker of ventilation heterogeneity) in 71 deployed individuals that was interpreted as evidence for a link between blast exposure and small airways injury. Whereas scoring instruments and standardized interviews exist to assess blast-related TBI and associated neuro-psychological sequelae beyond self-report [8, 9], the validity of these approaches have not been assessed in the context of adverse respiratory system effects.

This study examined whether pulmonary function was associated with clinician-documented blast exposure during deployment among a large cohort of deployed Veterans referred for specialty evaluation. We first developed a rigorous chart abstraction process and associated template with a multi-disciplinary team of clinicians and scientists. Clinical encounter notes were then reviewed to establish blast exposure case assignment with emphasis on TBI clinician notes as a proxy for physiological effect from blast exposure. Pulmonary function was then compared across blast exposure groups adjusting for confounding factors. We hypothesized that a history of blast exposure during deployment would be associated with impaired pulmonary function variables in a dose-dependent manner.

## Methods

### Sample description

The present cohort is comprised of combat deployed Veterans (n = 601) referred to our national post-deployment health clinic (New Jersey War Related Illness and Injury Study Center (NJ WRIISC) [10]) between 2011 and 2019 who underwent pulmonary function testing (PFT) as part of their multi-day clinical evaluation as previously described [11]. We limited the present analysis to those Veterans deployed in support of operations to Southwest Asia and Afghanistan starting in 2001 (n = 315). Additional exclusion criteria included subjects with deployment lengths less than one month or missing deployment history (n = 4). As this was a retrospective review of medical records that did not require contacting patients, a waiver of consent was obtained. Ethics approval was obtained from the VA New Jersey Health Care System Research & Development Committee (#01298).

### Clinical evaluations

Comprehensive evaluations performed by an interdisciplinary team were tailored to the Veteran yet consist of the following basic elements: (1) medical history review and physical examination, (2) occupational and environmental medicine history, (3) PFT with bronchodilator, (4) neuropsychological or psychological evaluation, and (5) standardized intake questionnaire packet. By design, Veterans referred to the NJ WRIISC endorse chronic symptoms that remain unexplained secondary to work-up at the Veteran’s home VA Medical Center. Depending on presenting symptoms, Veterans may also receive additional specialty testing as clinically indicated, including specialized pulmonary testing.

All Veterans, irrespective of chief complaint, underwent complete PFT in accordance with published guidelines [12] using commercially available equipment (Cosmed Quark PFT, Q-Box, i2M; Rome, Italy). PFT was performed in the morning in a semi-fasted state after an overnight withdrawal of bronchodilators (if applicable) as previously described [11]. Tests were performed in the following order: (1) spirometry, (2) lung volumes via body plethysmography, (3) diffusing capacity of carbon monoxide via the single-breath technique (DL_CO_), and (4) post-bronchodilator spirometry. Published reference equations were used for interpretation and reporting of spirometry [13], lung volumes [14], and DL_CO_ [15] (hemoglobin corrected [16]). Beginning in 2013, Veterans typically also underwent additional cardiopulmonary testing including the forced oscillation technique (FOT) before and after bronchodilator (400 µg salbutamol via spacer) as previously described [11].

Analysis of pulmonary function focused on the following outcomes from the pulmonary function tests: total lung capacity (TLC%; % predicted), forced expiratory volume in 1 s (FEV_1_%; % predicted), % change in FEV_1_ after bronchodilator (%∆FEV_1_PB), the FEV_1_ to forced vital capacity ratio (FEV_1_/FVC), the hemoglobin-corrected DL_CO_ (DL_CO_%, % predicted), frequency dependence of resistance (R4–R20%), % change in reactance area after bronchodilator (%∆AX), and % change in resistance and reactance at the lowest frequency (4 Hz) after bronchodilator (%∆R4PB and %∆X4PB). These variables were selected to provide broad representation of pulmonary flow, volume, diffusion, and mechanics.

### Initial blast exposure assessment and characterization

Aside from self-report (yes/no) to blast exposure as indicated on the intake questionnaire, Veterans did not undergo a routine and standardized assessment of blast exposure during their clinical evaluation. However, each Veteran underwent a one-on-one exposure evaluation with an occupational and environmental medicine physician or other trained physician during which blast and other exposures were specifically inquired about. Exposure to blast was also frequently discussed with a provider during other components of the clinical evaluation such as: (1) TBI screening conducted by a neuropsychologist or mental health provider, (2) history and physical conducted by a physician or nurse practitioner, and/or (3) cardiopulmonary evaluation by a pulmonologist. Text from the clinical notes of these encounters provided the source of information for characterizing blast exposure.

### Retrospective chart review to characterize blast exposure

An a priori chart abstraction process was designed by a multidisciplinary working group of NJ WRIISC clinicians and scientists with expertise in pulmonary medicine, internal medicine, environmental and occupational medicine, neuropsychology, and exercise physiology. Although several instruments are available to evaluate neuropsychological sequelae of TBI (blast-related and non-blast-related) (e.g., [8, 9]), the working group was unable to identify an existing instrument designed to assess the impact of blast exposure on the cardiopulmonary system. Therefore, we developed a process and tool for extracting key variables from the clinical notes to derive an assessment of blast exposure.

A chart reviewer template was developed to guide the chart abstraction process. This template consisted of preselected key variables: (1) proximity to blast (< 50 m, 50–100 m, 101–200 m, > 200 m); (2) number of sub-concussive and concussive blasts; (3) severity of acute symptoms for TBI caused by blast (absent, mild, moderate) [17]; (4) PTSD associated with blast (yes, maybe, no); (5) CDC blast injury definitions (primary: injury from blast pressure wave, secondary: injury from resultant projectiles, tertiary: injury from being moved by blast wind, quaternary: all other blast related injuries such as burns) [18]; (6) and whether or not the patient was physically moved by the blast (yes, no). One chart reviewer (clinician) read through each patient’s record to abstract responses for each key variable from the WRIISC clinicians’ documentation of blast exposures (n = 311). Two additional researchers (non-clinicians) reviewed and abstracted information from a random selection of 10% of the charts using the same template. Blinded to the initial reviewer’s results, this allowed for assessment of interrater reliability for the chart abstraction instrument.

Initially, reliability, completeness, and consistency of the clinical notes for the key variables of interest of the chart abstraction process were unclear; upon further examination of the abstracted data, the variables for number of concussive blasts and TBI severity were selected to define blast exposure groupings. These variables were selected because they were the most complete and they provided the most consistent blast exposure assessment according to the interrater agreement of the chart abstraction process (> 92% agreement; Additional file 1: Table S1). Blast exposure groupings were defined as follows: (1) none (no TBI symptoms nor concussive blasts identified), (2) single mild blast exposure (one concussive blast incident identified with mild TBI symptoms), and (3) multiple mild blast exposures (more than one concussive blast incident identified with mild TBI symptoms). Single and multiple moderate/severe blast exposures were defined in the same manner, except were based on moderate/severe TBI symptoms. For a thorough reporting of the interrater agreement results, see Additional file 1: Table S1.

### Comorbid conditions and health problems

Patient charts were reviewed to abstract the International Classification of Disease-9 and -10 (ICD9/ICD10) codes present in the WRIISC clinician’s notes. ICD9 codes were converted to ICD10 codes to organize into comorbid condition and health problem groupings [19]. Comorbid condition and health problem groupings were determined by a physician (DAH) consistent with ICD taxonomy. Patients were counted as having a comorbid condition and health problem if one or more constituent ICD code was present and we calculated the frequency of each comorbidity in the sample. All comorbid conditions and health problems with a prevalence > 10% in the study sample are reported.

### Data and statistical analysis

Assessments for differences among blast exposure groupings for patient characteristics were conducted with the Kruskal–Wallis test for continuous variables followed by Dunn test for post-hoc multiple comparisons. Fisher’s exact test was used to assess for association between categorical patient characteristics and blast grouping. The interrater reliability analysis for the chart abstraction was conducted using Gwet’s AC2 with linear weighting [20, 21]. A more comprehensive analysis using multivariable linear regression models to assess the effect of blast exposure on specific pulmonary function outcomes was also pursued and described in the Additional file 1. All analyses were conducted using the R software for statistical computing [22].

## Results

### Blast characteristics

After the chart abstraction process, all subjects were categorized according to blast group as follows: none (n = 208), single mild blast exposure (n = 52), and multiple mild blast exposures (n = 47) (Table 1). Due to a low representation of moderate/severe blast exposure in this dataset (n = 4), these subjects were excluded from further analyses. Specific, abstracted blast characteristics generally aligned well with assigned blast category. For example, the experience of being physically moved by the blast was far more common among those with “single mild” (44.2%) or “multiple mild” blasts (36.2%) compared to those with “no blast” (5%). Similarly, experiencing higher order blast effects (secondary, tertiary or quaternary effects according to the CDC classification [18]) was not applicable in 82.2% of those with no blast (and not documented in 12.0%), while everyone classified with one or more blast had documentation related to higher order blast effects and > 70% experienced one or more of these effects. All analyses presented below use the “no blast,” “single mild,” and “multiple mild” blast groups.

**Table 1:** Blast characteristics identified from chart abstraction process and resultant blast grouping assignments

Study assigned blast grouping	Overall (n = 307)		No blast (n = 208)		Single mild (n = 52)		Multiple mild (n = 47)	
Blast characteristics	n	%	n	%	n	%	n	%
Proximity to Blast								
Not applicable	153	49.8	140	67.3	7	13.5	6	12.8
< 50 m	97	31.6	24	11.5	40	76.9	33	70.2
50–100 m	19	6.2	12	5.8	1	1.9	6	12.8
101–200 m	7	2.3	5	2.4	2	3.8	0	0.0
> 200 m	19	6.2	15	7.2	2	3.8	2	4.3
Missing data	12	3.9	12	5.8	0	0.0	0	0.0
Number of sub-concussive blasts								
Not applicable	71	23.1	70	33.7	0	0.0	1	2.1
0	89	29.0	62	29.8	17	32.7	10	21.3
1	11	3.6	6	2.9	4	7.7	1	2.1
> 1	111	36.2	45	21.6	31	59.6	35	74.5
Missing data	25	8.1	25	12.0	0	0.0	0	0.0
Number of concussive blasts								
0	208	67.8	208	100.0	0	0.0	0	0.0
1	52	16.9	0	0.0	52	100.0	0	0.0
2	12	3.9	0	0.0	0	0.0	12	25.5
≥ 3	35	11.4	0	0.0	0	0.0	35	74.5
Missing data	0	0.0	0	0.0	0	0.0	0	0.0
TBI symptoms								
Absent	208	67.8	208	100.0	0	0.0	0	0.0
Mild	98	31.9	0	0.0	52	100.0	46	97.9
Moderate	1	0.3	0	0.0	0	0.0	1	2.1
Missing data	0	0.0	0	0.0	0	0.0	0	0.0
PTSD associated with blast								
Not applicable	31	10.1	24	11.5	4	7.7	3	6.4
Yes	26	8.5	9	4.3	12	23.1	5	10.6
Maybe	61	19.9	25	12.0	16	30.8	20	42.6
No	102	33.2	66	31.7	18	34.6	18	38.3
Missing data	87	28.3	84	40.4	2	3.8	1	2.1
Blast related symptoms or injury*								
Not applicable	198	64.5	171	82.2	15	28.8	12	25.5
Primary	30	9.8	6	2.9	11	21.2	13	27.7
Secondary	6	2.0	1	0.5	3	5.8	2	4.3
Tertiary	25	8.1	3	1.4	13	25.0	9	19.1
Quaternary	23	7.5	2	1.0	10	19.2	11	23.4
Missing data	25	8.1	25	12.0	0	0.0	0	0.0
Physically moved by blast								
Not applicable	89	29.0	88	42.3	0	0.0	1	2.1
Yes	45	14.7	5	2.4	23	44.2	17	36.2
No	147	47.9	89	42.8	29	55.8	29	61.7
Missing data	26	8.5	26	12.5	0	0.0	0	0.0

*According to CDC blast injury definitions [18]. *TBI* traumatic brain injury, *PTSD* posttraumatic stress disorder

### Patient characteristics

Patient characteristics are presented in Table 2. Overall, the mean group age was 40.5 ± 9.7 (mean ± SD) years, evaluated 8.6 ± 3.8 years after last deployment, mostly male, never or former smokers, and non-Hispanic white and with a mean body mass index of 30.4 ± 5.2 kg/m^2^. Median total deployment duration was found to be statistically significantly different across blast groups [H (Kruskal Wallis test statistic) = 6.05, p = 0.03] with the multiple mild blast exposure group having a significantly longer total deployment length as compared to the group with no blast exposure [Z (post hoc Dunn’s test statistic) = 2.28, p = 0.03]. Blast exposure group was also associated with military branch (p = 0.001) and sex (p = 0.03). None of the remaining characteristics were statistically significantly different across groups. Also presented in Table 2 are 21 different comorbid condition and health problem groupings with > 10% prevalence in the study sample. The most common were PTSD (62%) and dyspnea (55%); none of which were statistically significantly different across blast groups. Self-reported lower respiratory symptoms (scored as none (24.8%), mild (22.1%), moderate (15.6%), and severe (15.3%), percentages from the overall sample) were also similar across blast groups (p = 0.71).

**Table 2:** Patient characteristics, comorbid conditions and health problems across assigned blast groupings

	Overall (n = 307)		No blast (n = 208)		Single mild (n = 52)		Multiple mild (n = 47)		p-value***
	Mean	SD	Mean	SD	Mean	SD	Mean	SD	
Age (years)	40.5	9.7	41.0	9.8	39.4	9.4	39.2	9.2	0.25
Height (m)	1.8	0.1	1.7	0.1	1.8	0.1	1.8	0.1	0.11
Weight (kg)	93.8	18.9	92.4	18.1	98.2	16.0	94.8	24.2	0.13
BMI (kg/m^2^)	30.4	5.2	30.2	5.0	31.1	4.9	30.5	6.2	0.57
Cumulative deployment duration (months)	14.4	8.6	13.4	7.7	14.8	7.8	18.1	11.8	0.03
Post-deployment duration (years) (Missing data: n = 1, 0.3%)	8.6	3.8	8.4	3.9	8.9	3.5	8.8	3.4	0.74

	Median	IQR	Median	IQR	Median	IQR	Median	IQR	
Smoking pack years* (Missing data: n = 11, 3.6%)	0	0, 9	0	0, 9	0	0, 9	4	0, 10	0.08

	n	%	n	%	n	%	n	%	
Sex									
Male	257	83.7	166	79.8	48	92.3	43	91.5	0.03
Female	50	16.3	42	20.2	4	7.7	4	8.5	
Missing data	0	0.0	0	0.0	0	0.0	0	0.0	
Race/Ethnicity									
Non-Hispanic White	193	62.9	123	59.1	37	71.2	33	70.2	0.18
Non-Hispanic Black	19	6.2	15	7.2	1	1.9	3	6.4	
Non-Hispanic Other	5	1.6	4	1.9	1	1.9	0	0.0	
Hispanic	57	18.6	37	17.8	10	19.2	10	21.3	
Unknown	33	10.7	29	13.9	3	5.8	1	2.1	
Missing data	0	0.0	0	0.0	0	0.0	0	0.0	
Branch									
Army	203	66.1	128	61.5	35	67.3	40	85.1	0.001
Air Force	42	13.7	38	18.3	2	3.8	2	4.3	
Marine	39	12.7	21	10.1	13	25.0	5	10.6	
Navy	18	5.9	16	7.7	2	3.8	0	0.0	
Missing data	5	1.6	5	2.4	0	0.0	0	0.0	
Smoking status									
Never	150	48.9	109	52.4	27	51.9	14	29.8	0.06
Former	104	33.9	67	32.2	16	30.8	21	44.7	
Current	53	17.3	32	15.4	9	17.3	12	25.5	
Missing data	0	0.0	0	0.0	0	0.0	0	0.0	
Lower respiratory symptoms**									
None	76	24.8	51	24.5	16	30.8	9	19.1	0.71
Mild (1/3)	68	22.1	45	21.6	11	21.2	12	25.5	
Moderate (2/3)	48	15.6	35	16.8	6	11.5	7	14.9	
Severe (3/3)	47	15.3	31	14.9	6	11.5	10	21.3	
Missing data	68	22.1	46	22.1	13	25.0	9	19.1	
Comorbid conditions and health problems (> 10% prevalence)									
PTSD	190	61.9	125	60.1	35	67.3	30	63.8	0.81
Dyspnea	170	55.4	118	56.7	31	59.6	21	44.7	0.28
Other sleep problems (Insomnia, restless legs syndrome, etc.)	107	34.9	78	37.5	15	28.8	14	29.8	0.38
Hearing loss/tinnitus	101	32.9	65	31.3	24	46.2	12	25.5	0.07
Axial pain	95	30.9	65	31.3	15	28.8	15	31.9	0.95
Headache/migraine	91	29.6	58	27.9	19	36.5	14	29.8	0.65
Depression	82	26.7	55	26.4	15	28.8	12	25.5	0.92
Sleep apnea	81	26.4	48	23.1	20	38.5	13	27.7	0.08
Fibromyalgia	78	25.4	50	24.0	18	34.6	10	21.3	0.24
Irritable bowel syndrome	75	24.4	49	23.6	17	32.7	9	19.1	0.39
Extremity pain	72	23.5	47	22.6	11	21.2	14	29.8	0.53
Upper respiratory issues (Rhinitis, sinusitis, etc.)	70	22.8	48	23.1	12	23.1	10	21.3	0.98
Traumatic brain injury	63	20.5	44	21.2	9	17.3	10	21.3	0.89
Neuropathy	63	20.5	40	19.2	12	23.1	11	23.4	0.72
Cognitive problems	61	19.9	35	16.8	16	30.8	10	21.3	0.08
Fatigue	54	17.6	32	15.4	12	23.1	10	21.3	0.49
Vitamin D deficiency	47	15.3	31	14.9	7	13.5	9	19.1	0.57
Cough	42	13.7	28	13.5	10	19.2	4	8.5	0.31
Asthma	41	13.4	32	15.4	6	11.5	3	6.4	0.27
Gastroesophageal reflux disease	38	12.4	26	12.5	6	11.5	6	12.8	1.00
Other pain	35	11.4	24	11.5	6	11.5	5	10.6	1.00

*Due to non-normality, data are presented as median and interquartile range. **Self-reported cough, wheeze and/or dyspnea ≥ twice per week. ***To assess differences among blast groups, Kruskal–Wallis test followed by Dunn test for post hoc multiple comparisons was used for continuous variables while Fisher’s exact test was used for categorical variables. *BMI* body mass index, *PTSD* posttraumatic stress disorder

### Pulmonary function test findings

**Fig. 1:**
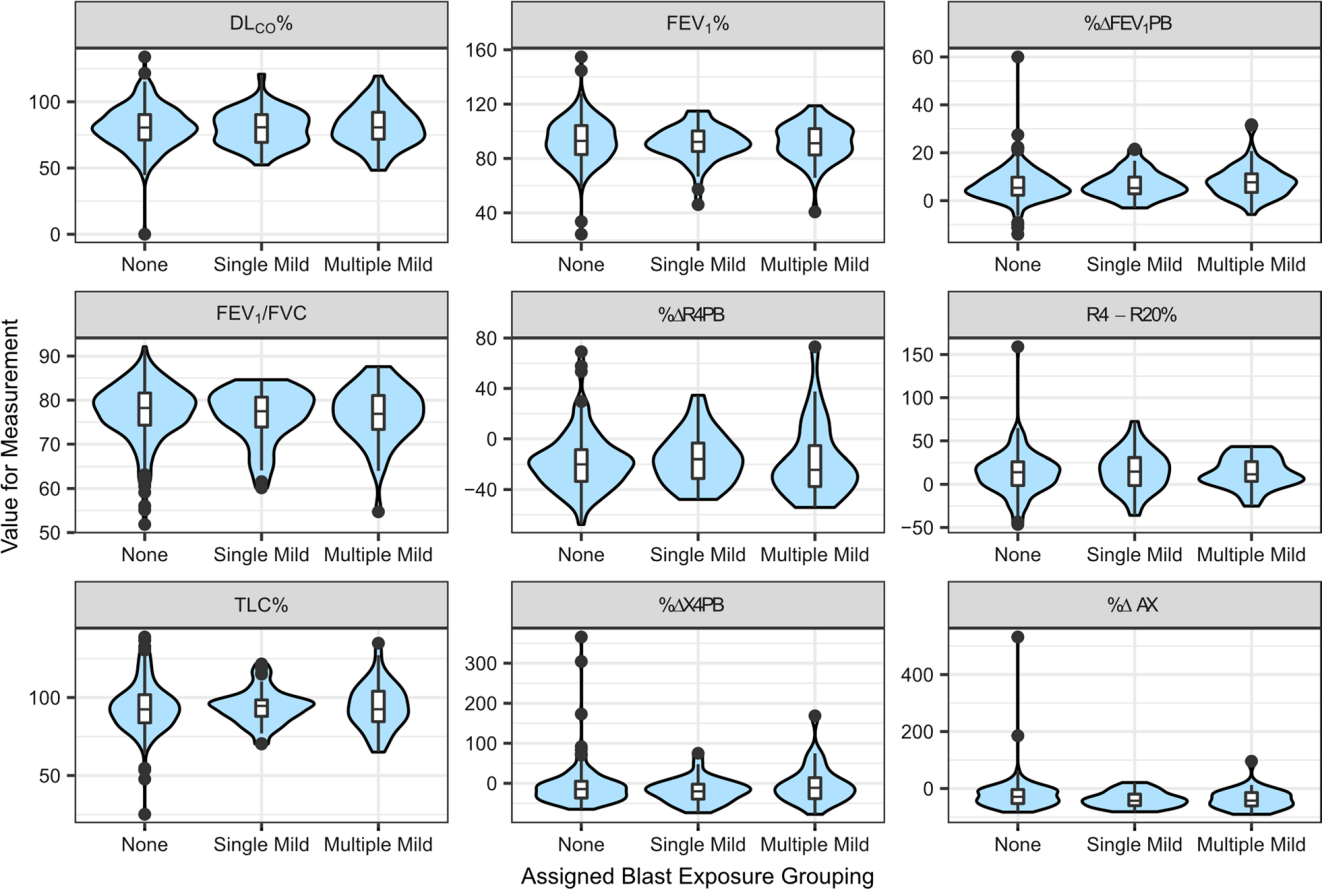
Violin plots of pulmonary function test findings across assigned blast exposure groups. Blast exposure group assignments are as follows: none (n = 208), single mild (n = 52), and multiple mild (n = 47). The y-axis represents the range for each of the nine respective variables and measurement units are dependent on variable type: Total Lung Capacity (TLC%; % predicted), Forced Expiratory Volume at 1 s (FEV_1_%; % predicted), % change in FEV_1_ after bronchodilator (%∆FEV1PB), the FEV_1_ to Forced Vital Capacity ratio (FEV_1_/FVC), the corrected DLCO (DL_CO_%, % predicted), difference in resistance between 4 and 20 Hz (R4–R20%), % change in reactance area after bronchodilator (%∆AX), and % change in resistance and reactance at the lowest frequency (4 Hz) after bronchodilator (%∆R4PB and %∆X4PB)

The overall trends and distribution of data, as indicated by the shape and location of the violin plots and inner boxplots, were similar across the three blast groups for each of the nine selected pulmonary function test outcomes (Fig. 1). To support and complement these qualitative findings, statistical analyses comparing these pulmonary function outcomes across blast groups are described and presented in the Additional file 1: Supplemental Statistical Analyses, Tables S2–S4. Overall, there were no differences across groups for any outcome measure, irrespective of the level of model adjustment (all p values 0.10–0.99 without correction for multiple comparisons). A more thorough reporting of results for additional parameters for lung volumes, diffusion, airflow, and FOT are included in Additional file 1: Tables S5–S7.

## Discussion

We hypothesized lung injury from mild blast exposure during deployment will result in impaired pulmonary function. In the absence of standardized instruments to classify blast exposure to the thorax, we first developed a standardized approach to characterize blast exposure derived from clinical interviews with emphasis on TBI as a proxy for physiological effect from blast exposure. After assigning Veterans to exposure groups (i.e., none, single- or multiple-mild), we evaluated whether group assignment was associated with select pulmonary function outcomes by comparing the overall trends and distribution of data (Fig. 1) as well as through statistical analyses (Additional file 1). Overall, with the current approach, we did not observe an association between clinician-documented blast exposure and objective measures of pulmonary function in this sample of deployed Veterans referred for specialty evaluation.

As highlighted by the National Academies of Sciences, Engineering, and Medicine, “…there is a striking absence of data on the long-term pulmonary outcomes of exposure to blast (pg. 138; [23]).” In a case series of 11 civilians who survived a bus terrorist explosion, most were reported to have normal cardiopulmonary function one year after their injury [24]. A direct comparison to the present study’s results is difficult given substantial differences in injury severity and length of follow-up. However, given that pulmonary function was similar across groups, the present study’s findings may be considered consistent with that of Hirshberg and colleagues [24]. Zell-Baran et al. [7] observed greater lung clearance index, suggestive of small airway injury, among previously deployed individuals (n = 71) with higher blast exposure intensity scores. This association was no longer significant after adjustment and no other pulmonary function parameters were considered. In the present analysis, we did not observe an association with blast exposure and any pulmonary function outcome, including multiple indices of small airway function via oscillometry. This inconsistency may be related to several factors, including the study design, as well as differences in blast exposure characterization. For example, in addition to IED blast exposures, Zell-Baran and colleagues included frequency of exposure to controlled detonations, which the present study was unable to ascertain. Looking beyond IED exposure is important as past research indicates that repetitive, low level blast exposures such as from routine training with weapons can lead to chronic cumulative pathophysiological effects [25, 26].

There are two major limitations with this study: (1) potential contributors to errors in blast exposure assessment, and (2) confounding due to underlying comorbid conditions and health problems. In assessing blast exposure, it is challenging to disentangle recollections of prior blast exposures versus experiences. Martindale et al. [27] highlighted that psychological stress associated with blast experiences (i.e., hearing/seeing a blast) during deployment mediates symptom reporting, potentially resulting in reporting of symptoms similar to TBI regardless of whether a TBI or other physical trauma has occurred [27, 28]. A second interrelated issue is the reliance on TBI as a proxy for blast overpressure wave impact on the pulmonary system. Recent evidence suggests that TBI symptomatology is not necessarily indicative of blast exposure severity [27], prompting calls to update TBI classification schemes to better align with physiological outcomes [23]. Third, patients’ recollections of blasts and/or clinical documentation may be biased towards more memorable blast experiences. Objective measurement of the blast-overpressure experienced by an individual will be the least biased assessment possible.

The observed lack of an association between blast exposure and objective measures of pulmonary function in the present analysis may also be attributable to various confounding factors intrinsic to the examination of a clinical sample. Foremost, Veterans referred to the NJ WRIISC have chronic, unexplained symptoms, including dyspnea and many other symptoms and deployment-related concerns. The presence of multiple and diverse comorbid conditions and health problems may have compromised the ability to detect the association between blast exposure and measure of long-term pulmonary function. Similarly, almost 20% of the sample were current smokers and about one third were former smokers albeit with a minimal pack-year history (Table 2). While we did control for smoking pack years and BMI in the adjusted models with no meaningful differences in results relative to unadjusted models, we did not control for comorbid conditions and health problems (Additional file 1). The use of past clinicians’ notes allowed us to assess the presence of comorbid conditions and health problems using ICD9/10 codes at one point in time, but the relationships among these comorbidities, blast exposure and pulmonary function were not clear. Future prospective studies should ask explicitly about the presence and onset of each comorbidity of interest to control for potential confounders.

This study exhibited strengths in its design and offers important insight for future research. This is a large well-described single site cohort evaluated by a multidisciplinary team of subspecialty clinicians obtained approximately 9 years after deployment. The high inter-rater reliability (> 92%) for the variables we abstracted from the medical record to define blast exposure are reflected in Table 1. Characteristics of blast exposure generally aligned well with concussive symptoms and blast experience. Because this work has illustrated the ability to consistently abstract relevant clinician notes, the key to improving future work will be improving accuracy of the interview for the targeted purpose assessing blast overpressure wave exposure to pulmonary system.

## Conclusions

In this retrospective analysis of medical records, Veterans deployed to Iraq and Afghanistan with or without clinician-documented mild blast exposure demonstrate similar pulmonary function. Reliance on clinical interviews tailored to evaluate blast-related TBI as a proxy for blast-related thoracic injury may have impacted our ability to differentiate between groups. However, our approach could be modified for future investigations designed to distinguish between blast *exposures* versus *experiences*. Moreover, the clinical findings and experience presented herein may also aid the design and development of prospective controlled studies to better characterize potential blast exposure persistent and latent effects on cardiopulmonary health.

## Supplementary Information

**Additional file 1.** Contains the following supplemental tables as described in the manuscript text: (1) **Table S1.** Key variables used for retrospective chart abstraction process and interrater reliability. (2) **Table S2.** Number of subjects in regression models and additional number (%) of subjects excluded in adjusted models due to incomplete predictors data. (3) **Table S3.** Model fit results by outcome measure and model type. (4) **Table S4.** Model fit results (effect estimate and (p-value)) by outcome measure and model adjustment for blast exposure group (any mild blast exposure) vs. reference group (no blast exposure). (5) **Table S5.** Additional pulmonary function test findings: lung volumes and diffusion. (6) **Table S6.** Additional pulmonary function test findings: spirometry. (7) **Table S7.** Additional pulmonary function test findings: forced oscillation technique.

## Data Availability

The datasets generated and/or analyzed during the current study are not publicly available or available in de-identified form as these our clinical data from electronic health records.

## Abbreviations

BMI: Body mass index
CDC: Centers for Disease Control and Prevention
CI: Confidence interval
DL_CO_: Diffusing capacity of carbon monoxide via the single-breath technique
FEV1: Forced expiratory volume in 1 s
FOT: Forced oscillation technique
FVC: Forced vital capacity
IED: Improvised explosive devices
ICD: International Classification of Diseases
IRR: Inter-rater reliability
NJ WRIISC: New Jersey War Related Illness and Injury Study Center
PB: Post bronchodilator
PTSD: Post-traumatic stress disorder
PFT: Pulmonary function test
TLC: Total lung capacity
TBI: Traumatic brain injury
VA: Veterans Affairs
