# Supplementary material for: Relationship between clinician documented blast exposure and pulmonary function: a retrospective chart review from a national specialty clinic
Source: Respir Res. 2022 Jun 10;23:153. doi: 10.1186/s12931-022-02071-0 (PMC9188057; doi:10.1186/s12931-022-02071-0)
Supplement: Supplementary file 1 — Additional file 1. Contains the following supplemental tables as described in the manuscript text: (1) Table S1. Key variables used for retrospective chart abstraction process and interrater reliability. (2) Table S2. Number of subjects in regression models and additional number (%) of subjects excluded in adjusted models due to incomplete predictors data. (3) Table S3. Model fit results by outcome measure and model type. (4) Table S4. Model fit results (effect estimate and (p-value)) by outcome measure and model adjustment for blast exposure group (any mild blast exposure) vs. reference group (no blast exposure). (5) Table S5. Additional pulmonary function test findings: lung volumes and diffusion. (6) Table S6. Additional pulmonary function test findings: spirometry. (7) Table S7. Additional pulmonary function test findings: forced oscillation technique. [file 12931_2022_2071_MOESM1_ESM.docx]

**Supplemental Material**

**Title:**

Relationship between clinician documented blast exposure and pulmonary function: A retrospective chart review from a national specialty clinic.

**Authors:**

Jennifer H. Therkorn^1^

Sean Hu^2^

Anays M. Sotolongo^1-2^

Israel C. Christie^3,4^

Tianshi David Wu^4,5^

William H. Van Doren^1^

Venkata Siva Sai Sujith Sajja^6^

Nisha Jani^1^

Jacquelyn C. Klein-Adams^1^

Drew A. Helmer*^3,4^

Michael J. Falvo*^1,2^

** Equal contribution*

**Affiliations**

^1^Airborne Hazards and Burn Pits Center of Excellence, War Related Illness and Injury Study Center, VA New Jersey Health Care System; East Orange, NJ, USA

^2^New Jersey Medical School, Rutgers – The State University; Newark, NJ, USA

^3^Section of Health Services Research, Baylor College of Medicine; Houston, TX, USA

^4^Center for Innovations in Quality, Effectiveness, and Safety, Michael E. DeBakey VA Medical Center; Houston, TX, USA

^5^Section of Pulmonary, Critical Care, and Sleep Medicine, Baylor College of Medicine; Houston, TX, USA

^6^Blast Induced Neurotrauma Branch, Walter Reed Army Institute of Research; Silver Spring, MD, USA

**Table S1. Key variables used for retrospective chart abstraction process and interrater reliability.**

| **Key Variables** | **Result Categories** | **Agreement (%) *** |
| --- | --- | --- |
| Proximity to Blast | <50m, 50-100m, 101-200m, >200m | 88 |
| Number of Sub-concussive Blast(s) | 0, 1, > 1 | 75 |
| Number of Concussive Blast(s) | 0, 1, 2, ≥ 3 | 95 |
| TBI Symptoms Based on TBI Literature | Absent, Mild, Moderate | 92 |
| PTSD Associated with Blast | Yes, No, Maybe | 74 |
| CDC Blast Related Symptoms/Injuries (Not from TBI) [1] | Primary, Secondary, Tertiary, Quaternary | 84 |
| Physically Moved by Blast | Yes, No | 69 |

Notes: * Results are presented as observed interrater percent agreement across three independent chart reviewers for a subset of the total number of charts (10%). The interrater reliability analysis was conducted using Gwet’s AC2 with linear weighting.

**Supplemental statistical analyses**

To complement the qualitative analysis provided in the main manuscript, we further tested the hypothesis that blast exposure was associated with the selected pulmonary function outcomes. For each outcome measure, a simple and multivariable linear regression model were fit to assess for statistically significant difference in blast grouping. The simple model used blast grouping as the only predictor. To statistically control for potential group differences acting as confounders, the adjusted multivariable model included the following covariates: age, gender, height, weight, race/ethnicity, smoking pack years, post deployment length, body mass index (BMI), total deployment length and service branch. For the adjusted versus unadjusted models, any subject with incomplete predictors data were handled with listwise deletion (complete case analysis), which was fewer than 5% of all subjects in each model (Supplemental Table S2). Each model’s residual diagnostic plots were checked to ensure they were normally distributed with constant variance and no apparent trends (residuals vs. fits, normal probability, and scale-location). Cook’s distance was used to confirm there were no potentially influential data points on the regression lines [2].

**Table S2. Number of subjects in regression models and additional number (%) of subjects excluded in adjusted models due to incomplete predictors data.**

| **Model (Response Variable)** | **Number of Subjects in Unadjusted Model (Subjects with Complete Data for Blast Grouping and Outcome Measure)** | **Number of Subjects in Adjusted Model (Subjects with Complete Data for Blast Grouping, Outcome Measure and Covariates)** | **Number (%) of Subjects Excluded in Adjusted Models (Incomplete Predictors Data) *** |
| --- | --- | --- | --- |
| TLC% | 298 | 288 | 10 (3.0) |
| FEV1% | 305 | 293 | 12 (3.9) |
| %∆FEV1PB | 297 | 286 | 11 (3.7) |
| FEV_1_/FVC | 305 | 293 | 12 (3.9) |
| DL_CO_% | 283 | 274 | 9 (3.2) |
| %∆R4PB | 173 | 169 | 4 (2.3) |
| R4-R20% | 176 | 172 | 4 (2.3) |
| %∆AX | 171 | 167 | 4 (2.3) |
| %∆X4PB | 173 | 169 | 4 (2.3) |

Notes: * For the adjusted vs. unadjusted models, any subject with incomplete predictors data were handled with listwise deletion to allow for complete case analysis. Variables include the following: Total Lung Capacity (TLC%; % predicted), Forced Expiratory Volume at 1 second (FEV_1_%; % predicted), % change in FEV_1_ after bronchodilator (%∆FEV1PB), the FEV_1_ to Forced Vital Capacity ratio (FEV_1_/FVC), the corrected DLCO (DL_CO_%, % predicted), difference in resistance between 4 Hz and 20 Hz (R4-R20%), % change in reactance area after bronchodilator (%∆AX), and % change in resistance and reactance at the lowest frequency (4 Hz) after bronchodilator (%∆R4PB and %∆X4PB).

**Table S3. Model fit results by outcome measure and model type.**

| **Model Type** | **Simple (Unadjusted)** | | **Multivariable (Adjusted)** | |
| --- | --- | --- | --- | --- |
| **Model Outcome & Blast Group** | **Single Mild Blast** | **Multiple Mild Blasts** | **Single Mild Blast** | **Multiple Mild Blasts** |
| TLC% | 0.11 (0.99) | 0.44 (0.87) | -0.73 (0.77) | -0.70 (0.79) |
| FEV_1_% | -2.26 (0.36) | -1.53 (0.54) | -2.86 (0.30) | -1.62 (0.57) |
| %∆FEV_1_PB | 0.41 (0.72) | 1.90 (0.10) | 0.30 (0.71) | 2.13 (0.10) |
| FEV_1_/FVC | -0.77 (0.45) | -0.46 (0.67) | -0.10 (0.92) | 0.13 (0.91) |
| DL_CO_% | -0.26 (0.92) | 0.62 (0.82) | -0.62 (0.85) | 0.98 (0.73) |
| R4-R20% | 3.02 (0.54) | 0.85 (0.87) | 4.27 (0.41) | 1.09 (0.85) |
| %∆AX | 2.62 (0.31) | 2.15 (0.44) | 3.40 (0.19) | 2.72 (0.35) |
| %∆R4PB | 2.32 (0.63) | 0.44 (0.93) | 1.60 (0.75) | 3.18 (0.57) |
| %∆X4PB | -13.35 (0.22) | 2.33 (0.84) | -14.32 (0.21) | 3.51 (0.79) |

Notes: Model fit results are presented as effect estimate (p-value). The blast exposure group results are relative to the regression model reference group (no blast exposure). Model outcome variables include the following: Total Lung Capacity (TLC%; % predicted), Forced Expiratory Volume at 1 second (FEV_1_%; % predicted), % change in FEV_1_ after bronchodilator (%∆FEV1PB ), the FEV_1_ to Forced Vital Capacity ratio (FEV_1_/FVC), the corrected DL_CO_ (DL_CO_%, % predicted), difference in resistance between 4 Hz and 20 Hz (R4-R20%), % change in reactance area after bronchodilator (%∆AX ), and % change in resistance and reactance at the lowest frequency (4 Hz) after bronchodilator (%∆R4PB and %∆X4PB).

Table S3 presents the model fit results across the selected outcome measures for the simple and multivariable (i.e., covariate adjusted) fitted models. Effect estimates here represent the average change in response for those in the blast exposure groups as compared to the no blast exposure group, with all other covariates in model held constant (if applicable). Regardless of level of model adjustment, neither the single mild blast exposure nor multiple mild blast exposure groups were statistically significantly different from the no blast exposure group across all outcome measures (all p values 0.10-0.99 without correction for multiple comparisons). Only the %FEV_1_ post-bronchodilator metric demonstrated a possible ‘dose response’ pattern, although the effect sizes were not statistically significant. In the simple model, the effect size for single mild blast group was higher than no blast group (0.41; p=0.72) and even higher (1.90; p=0.10) in the multiple mild blast group. Given that effect size here represents the average response variable contribution associated with being in each of the assigned blast groups, one may interpret these results as illustrating a potential increase in the %FEV_1_ post-bronchodilator metric of 0.41 in comparing the multiple mild blast group to no blast group versus a potential increase of 1.90 in comparing the multiple moderate blast group to no blast group. This pattern was even more pronounced in the adjusted model (single mild effect size 0.30 compared to no blast (p=0.71); multiple mild blast effect size 2.13 (p=0.10)). Further investigation was performed by merging the single and multiple mild blast exposure groups together to understand the predicted differences in outcome measures versus the no blast exposure group. There were no statistically significant differences identified with all p values ranging from 0.14-0.99 (Table S4 below).

**Table S4. Model fit results (effect estimate and (p-value)) by outcome measure and model adjustment for blast exposure group (any mild blast exposure) vs. reference group (no blast exposure).**

| **Model Type** | **Simple (Unadjusted)** | **Multivariable (Adjusted)** |
| --- | --- | --- |
| **Model Outcome & Blast Group** | **Any Mild Blast Exposure** | **Any Mild Blast Exposure** |
| TLC% | 0.27 (0.89) | -0.72 (0.72) |
| FEV_1_% | -1.91 (0.32) | -2.23 (027) |
| %∆FEV_1_PB | 1.13 (0.19) | 1.12 (0.26) |
| FEV_1_/FVC | -0.62 (0.44) | 0.003 (0.99) |
| DL_CO_% | 0.18 (0.93) | 0.13 (0.95) |
| R4-R20% | 2.01 (0.61) | 2.87 (0.49) |
| %∆AX | 2.41 (0.24) | 3.10 (0.14) |
| %∆R4PB | 1.47 (0.70) | 2.28 (0.58) |
| %∆X4PB | -6.20 (0.47) | -6.68 (0.48) |

Notes: Model outcome variables include the following: Total Lung Capacity (TLC%; % predicted), Forced Expiratory Volume at 1 second (FEV_1_%; % predicted), % change in FEV_1_ after bronchodilator (%∆FEV1PB ), the FEV_1_ to Forced Vital Capacity ratio (FEV_1_/FVC), the corrected DL_CO_ (DL_CO_%, % predicted), difference in resistance between 4 Hz and 20 Hz (R4-R20%), % change in reactance area after bronchodilator (%∆AX ), and % change in resistance and reactance at the lowest frequency (4 Hz) after bronchodilator (%∆R4PB and %∆X4PB).

**Additional parameters for lung volumes, diffusion, airflow and forced oscillometry**

**Table S5. Additional pulmonary function test findings: lung volumes and diffusion**

| **Measurement** | **n** | **Missing Data (n)** | **Observed** | | **% Predicted** | |
| --- | --- | --- | --- | --- | --- | --- |
|  |  |  | **Mean** | **SD** | **Mean** | **SD** |
| **Overall** | **307** |  |  |  |  |  |
| TLC | 298 | 9 | 6.30 | 1.26 | 94.07 | 15.13 |
| RV | 298 | 9 | 1.51 | 0.69 | 78.81 | 33.25 |
| FRC | 299 | 8 | 3.07 | 0.93 | 92.12 | 26.21 |
| RV/TLC | 298 | 9 | 24.28 | 11.75 | 79.77 | 35.31 |
| FRC/TLC | 299 | 8 | 48.91 | 12.88 | 93.89 | 24.50 |
| DL_CO_ | 283 | 24 | 25.61 | 5.84 | 80.67 | 15.71 |
| **No Blast** | **208** |  |  |  |  |  |
| TLC | 203 | 5 | 6.21 | 1.30 | 93.99 | 16.06 |
| RV | 204 | 4 | 1.50 | 0.68 | 78.40 | 33.49 |
| FRC | 204 | 4 | 3.03 | 0.92 | 91.73 | 25.89 |
| RV/TLC | 204 | 4 | 24.69 | 12.94 | 80.17 | 38.70 |
| FRC/TLC | 204 | 4 | 49.26 | 13.96 | 94.33 | 26.39 |
| DL_CO_ | 194 | 14 | 25.13 | 5.97 | 80.62 | 16.29 |
| **Single Mild Blast** | **52** |  |  |  |  |  |
| TLC | 49 | 3 | 6.60 | 1.12 | 94.10 | 11.25 |
| RV | 48 | 4 | 1.53 | 0.69 | 77.46 | 28.24 |
| FRC | 49 | 3 | 3.09 | 0.89 | 90.00 | 22.70 |
| RV/TLC | 48 | 4 | 23.02 | 8.52 | 77.06 | 24.21 |
| FRC/TLC | 49 | 3 | 46.49 | 8.76 | 89.82 | 16.70 |
| DL_CO_ | 45 | 7 | 26.67 | 5.14 | 80.36 | 13.50 |
| **Multiple Mild Blasts** | **47** |  |  |  |  |  |
| TLC | 46 | 1 | 6.37 | 1.21 | 94.42 | 14.74 |
| RV | 46 | 1 | 1.55 | 0.73 | 82.04 | 37.30 |
| FRC | 46 | 1 | 3.21 | 1.03 | 96.14 | 30.87 |
| RV/TLC | 46 | 1 | 23.80 | 8.83 | 80.80 | 29.17 |
| FRC/TLC | 46 | 1 | 49.88 | 11.38 | 96.16 | 22.50 |
| DL_CO_ | 44 | 3 | 26.67 | 5.78 | 81.24 | 15.47 |
| **Moderate/Severe*** | 4 |  |  |  |  |  |
| TLC | 4 | 0 | 5.24 | 1.76 | 87.8 | 18.8 |
| RV | 4 | 0 | 1.00 | 0.55 | 60.5 | 31.5 |
| FRC | 4 | 0 | 2.19 | 1.18 | 70.9 | 33.6 |
| RV/TLC | 4 | 0 | 18.56 | 3.76 | 65.7 | 18.0 |
| FRC/TLC | 4 | 0 | 40.22 | 9.12 | 79.0 | 19.7 |
| DL_CO_ | 4 | 0 | 25.09 | 6.31 | 82.2 | 11.2 |

Notes: Measurement variables include the following: total lung capacity (TLC, L), residual volume (RV, L), functional residual capacity (FRC, L), diffusing capacity for carbon monoxide (DL_CO_, %) corrected for hemoglobin.

*Veterans with moderate (n = 3) or severe (n = 1) were excluded from our primary analyses.

**Table S6. Additional pulmonary function test findings: spirometry**

| **Measurement** | **n** | **Missing Data (n)** | **Observed** | | **% Predicted** | | **n** | **Missing** | **Post-Bronchodilator** | |
| --- | --- | --- | --- | --- | --- | --- | --- | --- | --- | --- |
|  |  |  | **Mean** | **SD** | **Mean** | **SD** |  |  | **Mean** | **SD** |
| **Overall** | 307 |  |  |  |  |  | 307 |  |  |  |
| FEV_1_ | 306 | 1 | 3.66 | 0.83 | 92.63 | 15.43 | 297 | 10 | 6.73 | 7.03 |
| FVC | 306 | 1 | 4.76 | 1.05 | 96.32 | 14.65 | 297 | 10 | 2.44 | 5.52 |
| FEV_1_/FVC | 305 | 2 | 77.07 | 6.48 | 96.02 | 8.33 | 297 | 10 | 80.20 | 6.05 |
| FEF_25-75_ | 306 | 1 | 3.38 | 1.12 | 89.22 | 28.16 | 296 | 11 | 18.41 | 18.48 |
| **No Blast** | 208 |  |  |  |  |  | 208 |  |  |  |
| FEV_1_ | 208 | 0 | 3.62 | 0.89 | 93.23 | 16.18 | 202 | 6 | 6.37 | 7.21 |
| FVC | 208 | 0 | 4.69 | 1.11 | 96.66 | 15.19 | 202 | 6 | 2.23 | 5.54 |
| FEV_1_/FVC | 208 | 0 | 77.26 | 6.58 | 96.19 | 8.29 | 202 | 6 | 80.35 | 6.13 |
| FEF_25-75_ | 208 | 0 | 3.37 | 1.18 | 90.04 | 28.29 | 201 | 7 | 17.98 | 18.92 |
| **Single Mild Blast** | 52 |  |  |  |  |  | 52 |  |  |  |
| FEV_1_ | 51 | 1 | 3.80 | 0.67 | 90.97 | 13.31 | 49 | 3 | 6.78 | 5.72 |
| FVC | 51 | 1 | 4.98 | 0.96 | 95.36 | 13.76 | 49 | 3 | 2.38 | 4.46 |
| FEV_1_/FVC | 50 | 2 | 76.50 | 6.13 | 95.39 | 8.20 | 49 | 3 | 79.69 | 5.96 |
| FEF_25-75_ | 51 | 1 | 3.42 | 0.95 | 86.48 | 26.35 | 49 | 3 | 17.55 | 16.11 |
| **Multiple Mild Blasts** | 47 |  |  |  |  |  | 47 |  |  |  |
| FEV_1_ | 47 | 0 | 3.69 | 0.70 | 91.70 | 14.19 | 46 | 1 | 8.27 | 7.40 |
| FVC | 47 | 0 | 4.81 | 0.88 | 95.87 | 13.28 | 46 | 1 | 3.47 | 6.41 |
| FEV_1_/FVC | 47 | 0 | 76.80 | 6.47 | 95.97 | 8.79 | 46 | 1 | 80.04 | 5.88 |
| FEF_25-75_ | 47 | 0 | 3.40 | 1.09 | 88.49 | 29.77 | 46 | 1 | 21.20 | 19.04 |
| **Moderate/Severe*** | 4 |  |  |  |  |  | 4 |  |  |  |
| FEV_1_ | 4 | 0 | 3.39 | 0.84 | 93.9 | 14.5 | 4 | 0 | 3.55 | 0.98 |
| FVC | 4 | 0 | 4.05 | 1.11 | 91.1 | 13.9 | 4 | 0 | 4.16 | 1.21 |
| FEV_1_/FVC | 4 | 0 | 84.08 | 4.22 | 103.5 | 7.23 | 4 | 0 | 85.5 | 3.2 |
| FEF_25-75_ | 4 | 0 | 3.84 | 1.02 | 107.2 | 37.7 | 4 | 0 | 4.42 | 1.25 |

Notes: Measurement variables include the following: forced expiratory volume in 1 second (FEV_1_, L), forced vital capacity (FVC, L), forced expiratory flow at 25% and 75% of the pulmonary volume (FEF_25-75_, L/s)

*Veterans with moderate (n = 3) or severe (n = 1) were excluded from our primary analyses.

**Table S7. Additional pulmonary function test findings: forced oscillation technique.**

| **Measurement** | **n** | **Missing Data (n)** | **Observed** | | **% Predicted** | | **n** | **Missing Data (n)** | **Post Bronchodilator** | |
| --- | --- | --- | --- | --- | --- | --- | --- | --- | --- | --- |
|  |  |  | **Mean** | **SD** | **Mean** | **SD** |  |  | **Mean** | **SD** |
| **Overall** | **307** |  |  |  |  |  | 307 |  |  |  |
| R4 | 175 | 132 | 4.29 | 1.72 | 128.38 | 48.99 | 174 | 133 | -17.49 | 23.69 |
| R20 | 177 | 130 | 3.44 | 1.00 | 118.19 | 37.75 | 174 | 133 | -12.50 | 15.20 |
| R4-R20 | 176 | 131 | 0.86 | 1.26 | NA | NA | 173 | 134 | -5.04 | 18.07 |
| X4 | 175 | 132 | -2.14 | 1.13 | 149.39 | 80.34 | 173 | 134 | -6.86 | 53.19 |
| AX | 171 | 136 | 13.24 | 12.66 | 311.08 | 289.11 | 165 | 142 | -25.81 | 56.75 |
| Fres | 177 | 131 | 3.37 | 1.50 | 133.73 | 51.42 | 171 | 136 | -7.89 | 43.22 |
| **No Blast** | 208 |  |  |  |  |  | 208 |  |  |  |
| R4 | 117 | 91 | 4.27 | 1.76 | 126.33 | 47.74 | 116 | 92 | -17.97 | 23.09 |
| R20 | 119 | 89 | 3.41 | 0.88 | 116.92 | 36.44 | 117 | 91 | -12.90 | 14.49 |
| R4-R20 | 118 | 90 | 0.87 | 1.39 | NA | NA | 116 | 92 | -5.14 | 18.29 |
| X4 | 117 | 91 | -2.09 | 0.97 | 144.78 | 69.64 | 116 | 92 | -4.81 | 58.08 |
| AX | 114 | 94 | 12.44 | 12.20 | 277.74 | 240.15 | 109 | 99 | -20.20 | 65.11 |
| Fres | 119 | 90 | 3.33 | 1.50 | 127.72 | 48.82 | 115 | 93 | -9.49 | 29.34 |
| **Single Mild Blast** | 52 |  |  |  |  |  | 52 |  |  |  |
| R4 | 31 | 21 | 4.20 | 1.69 | 127.30 | 49.42 | 31 | 21 | -15.65 | 20.46 |
| R20 | 31 | 21 | 3.40 | 1.18 | 117.07 | 38.55 | 31 | 21 | -10.51 | 13.30 |
| R4-R20 | 31 | 21 | 0.80 | 0.90 | NA | NA | 31 | 21 | -5.14 | 17.58 |
| X4 | 31 | 21 | -2.40 | 1.64 | 171.44 | 114.07 | 31 | 21 | -18.17 | 31.70 |
| AX | 31 | 21 | 15.06 | 15.00 | 374.89 | 391.57 | 31 | 21 | -37.34 | 27.74 |
| Fres | 31 | 21 | 3.48 | 1.81 | 142.15 | 58.60 | 31 | 21 | -12.75 | 16.11 |
| **Multiple Mild Blasts** | 47 |  |  |  |  |  | 47 |  |  |  |
| R4 | 27 | 20 | 4.49 | 1.67 | 138.58 | 54.33 | 26 | 21 | -17.53 | 30.01 |
| R20 | 27 | 20 | 3.60 | 1.27 | 125.08 | 42.97 | 26 | 21 | -13.06 | 20.14 |
| R4-R20 | 27 | 20 | 0.89 | 1.07 | NA | NA | 26 | 21 | -4.48 | 18.32 |
| X4 | 27 | 20 | -2.02 | 1.06 | 144.22 | 76.80 | 26 | 21 | -2.48 | 50.42 |
| AX | 26 | 21 | 14.59 | 11.76 | 382.46 | 330.56 | 25 | 22 | -35.95 | 39.27 |
| Fres | 27 | 20 | 3.38 | 1.11 | 150.51 | 50.76 | 25 | 22 | 5.02 | 91.47 |
| **Moderate/Severe*** | 4 |  |  |  |  |  | 4 |  |  |  |
| R4 | 1 | 3 | 8.06 | - | 147.79 | - | 1 | 3 | 2.88 | - |
| R20 | 1 | 3 | 5.50 | - | 123.9 |  | 1 | 3 | 4.05 | - |
| R4-R20 | 1 | 3 | 2.56 | - | NA | NA | 1 | 3 | -1.18 | - |
| X4 | 1 | 3 | -2.81 | - | 116.5 | - | 1 | 3 | -0.81 | - |
| AX | 1 | 1 | 40.79 | - | 341.7 | - | 1 | 1 | 5.68 | - |
| Fres | 1 | 1 | 31.56 | - | 182.7 |  | 1 | 1 | 15.42 | - |

Notes: Measurement variables include the following: low and mid frequency resistance (R4, R20, cmH_2_O·s/L), low and mid frequency resistance difference (R4-R20, cmH_2_O·s/L), low frequency reactance (X4, cmH_2_O·s/L), reactance area (AX, cmH_2_O/L), and resonant frequency (Fres, Hz)

*Veteran with moderate blast exposure (n = 1) excluded from our primary analyses.

**References**

1. **Explosions and blast injuries: a primer for clinicians** [<https://stacks.cdc.gov/view/cdc/28987>]

2. Cook RD: **Detection of Influential Observation in Linear Regression.** *Technometrics* 1977, **19:**15-18.
